# Biomaterials and Scaffold Design Strategies for Regenerative Endodontic Therapy

**DOI:** 10.3389/fbioe.2019.00317

**Published:** 2019-11-15

**Authors:** Gavin Raddall, Isabel Mello, Brendan M. Leung

**Affiliations:** ^1^Faculty of Dentistry, Dalhousie University, Halifax, NS, Canada; ^2^Department of Dental Clinical Sciences, Faculty of Dentistry, Dalhousie University, Halifax, NS, Canada; ^3^Department of Applied Oral Sciences, Faculty of Dentistry, Dalhousie University, Halifax, NS, Canada; ^4^School of Biomedical Engineering, Faculties of Medicine and Engineering, Dalhousie University, Halifax, NS, Canada

**Keywords:** biomaterials, bone, regenerative medicine, instructive scaffolds, endodontic therapy, stem cells, clinical considerations, blood-biomaterials interactions

## Abstract

Challenges with traditional endodontic treatment for immature permanent teeth exhibiting pulp necrosis have prompted interest in tissue engineering approaches to regenerate the pulp-dentin complex and allow root development to continue. These procedures are known as regenerative endodontic therapies. A fundamental component of the regenerative endodontic process is the presence of a scaffold for stem cells from the apical papilla to adhere to, multiply and differentiate. The aim of this review is to provide an overview of the biomaterial scaffolds that have been investigated to support stem cells from the apical papilla in regenerative endodontic therapy and to identify potential biomaterials for future research. An electronic search was conducted using Pubmed and Novanet databases for published studies on biomaterial scaffolds for regenerative endodontic therapies, as well as promising biomaterial candidates for future research. Using keywords “regenerative endodontics,” “scaffold,” “stem cells” and “apical papilla,” 203 articles were identified after duplicate articles were removed. A second search using “dental pulp stem cells” instead of “apical papilla” yielded 244 articles. Inclusion criteria included the use of stem cells from the apical papilla or dental pulp stem cells in combination with a biomaterial scaffold; articles using other dental stem cells or no scaffolds were excluded. The investigated scaffolds were organized in host-derived, naturally-derived and synthetic material categories. It was found that the biomaterial scaffolds investigated to date possess both desirable characteristics and issues that limit their clinical applications. Future research investigating the scaffolds presented in this article may, ultimately, point to a protocol for a consistent, clinically-successful regenerative endodontic therapy.

## Introduction

### Traditional Root Canal Treatment in the Context of Regenerative Endodontic Therapy

Major challenges are associated with current endodontic treatment of permanent teeth with pulpal necrosis and immature root development, found primarily in children and adolescent patients. Developing roots have open apices and thin dentinal walls resulting in fragility, which complicate the use of mechanical means to disinfect the root canal system (Friedlander et al., 2009; Lovelace et al., 2011). Consequently, endodontic therapies involving these teeth often rely on irrigation and intracanal medications to disinfect the canal space. Immature teeth that require endodontic treatment have been traditionally treated with long-term calcium hydroxide (Ca(OH)_2_). After initial disinfection of the pulp space, a calcium hydroxide paste is left in the canal to induce the deposition of a hard tissue barrier at the apical area. This barrier helps contain the filling materials in the canal without great risk of extravasation to the periapical tissues. This can be a lengthy process as it may take several months for the tissue barrier to form (Raldi et al., 2009). Alternatively, a more novel approach is the use of calcium silicate-based cements (MTA-like cements) to form an artificial apical plug with clinical outcomes superior to induced apexification by Ca(OH)_2_.

Both apexification techniques form a barrier on which permanent root canal filling material can be compacted against and promote healing of apical tissues. Apexification procedures do not promote further root development and the tooth will continue to have thin, fragile root canal walls that makes these teeth susceptible to cervical fracture from normal mastication forces or trauma (Wilkinson et al., 2007; Cotti et al., 2008). A clinical study conducted by Cvek (1992) demonstrated that the incidence of cervical root fracture ranged from 28 to 77% in immature teeth that had been treated with Ca(OH)_2_; teeth in earlier stages of development occupied the highest percentiles and were significantly more likely to fracture than mature teeth. Consequently, an alternative treatment protocol that can potentially reinforce the root and strengthen the root against fracture would help preserve the integrity of the afflicted tooth and maintain desirable function for patients.

**Figure 1:**
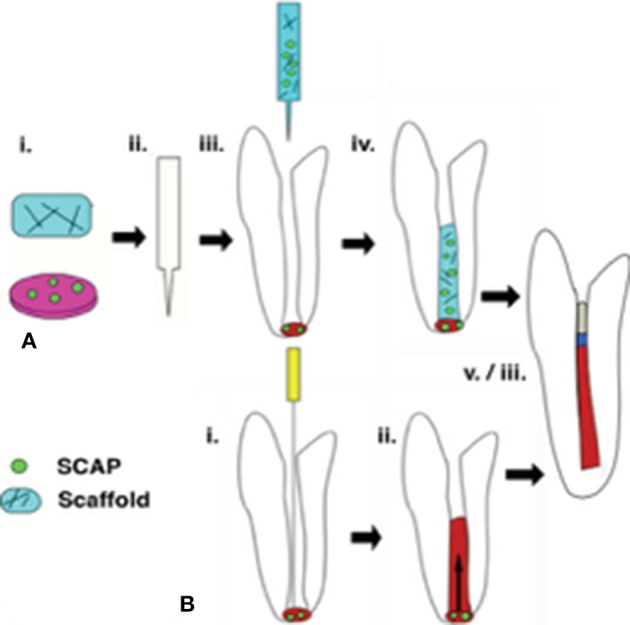
Regenerative endodontic therapies employ SCAP in achieving revascularization of the root canal and continued root development. **(A)** Following conservative preparation of the root canal and disinfection, cultured SCAP maybe combined with an injectable scaffold and inserted into the canal space. **(B)** The current clinical standard for regenerative therapies involves over instrumentation with and endodontic file to induce blood flow from the apical region into the canal space, forming a blood clot. SCAP migrate into the root canal as a result of this process. In a successful retreatment pathway. **(A,B)** both lead to root lengthening and dentinal wall thickening, as well as pulpal revascularization beneath an MTA seal and restoration material.

Regenerative endodontic therapy (RET) employs principles of bioengineering and is a contemporary alternative to conventional apexification procedures (Figure 1). It consists of irrigating the root canal space with low-concentration sodium hypochlorite (NaOCl) to dissolve necrotic tissue and disinfect the canal space, followed by the placement of Ca(OH)_2_ or an antibiotic mixture in the canal at the conclusion of the first appointment to further disinfect the dentinal tissue and protect the tooth from reinfection (Lee et al., 2015). In a subsequent appointment, the Ca(OH)_2_ or antibiotic mixture is removed, and a hand file is extended approximately 3 mm beyond the apical foramen to induce bleeding (Lee et al., 2015). Real-time reverse transcription polymerase chain reaction (rtPCR) and histologic evaluation of intracanal blood samples by Lovelace et al. demonstrated that mesenchymal stem cells (MSCs) are delivered to the root canal space after the induction of bleeding based on the expression of MSC markers CD105 and STRO-1; they speculated that these cells are ultimately responsible for deposition of both connective and hard tissues (Lovelace et al., 2011). After clot formation, a collagen matrix is placed at the cervical portion of the canal which is then sealed with MTA, followed by the placement of a bonded restoration (Lee et al., 2015). Over the ensuing 2 years, radiographic evidence is used to monitor root development along with a clinical examination (Lee et al., 2015). Although this is a viable, less complicated treatment alternative to traditional procedures, the outcomes of current revascularization strategies are often difficult to predict and an optimized protocol remains to be developed (Lee et al., 2015).

### Stem Cells From the Apical Papilla

Within the past decade, a population of postnatal mesenchymal stem cells from the apical papilla (SCAP) has been identified by Sonoyama et al. (2006) immediately adjacent to the root apex of immature teeth. Given the proximity of SCAP to the apical foramen, it has been suggested that these are the cells that enter the root canal space in current regenerative procedures (Lovelace et al., 2011). Importantly, the apical location of SCAP enables these cells to be supplied with collateral blood circulation, allowing them to survive during pulpal infection and necrosis (Huang et al., 2008). In both *in vitro* and *in vivo* analyses, it has been consistently observed that SCAP have the ability to differentiate into odontoblast-like cells that produce dentin in the root canal (Huang et al., 2009) While it has been determined that SCAP also have the capacity to undergo adipogenic and neurogenic differentiation *in vitro*, the same observations have not been made *in vivo* and it has been concluded that SCAP differentiate to only dentinogenic cells under *in vivo* conditions (Huang et al., 2009). Numerous biologically-active growth factors are trapped in the dentinal matrix during dentinogenesis, namely transforming growth factor-beta 1 (TGF- β1) and bone morphogenetic protein 2 (BMP-2), which are key in driving the odontogenic differentiation of SCAP, as well as vasoendothelial growth factor (VEGF), platelet-derived growth factor (PDGF) and other angiogenic factors that drive vascularization and pulpal regeneration (Zein et al., 2019).

Compared to other dental stem cells, SCAP are more capable of surviving infections such as apical periodontitis and abscesses, and have a superior ability to differentiate into dentin-forming cells (Huang et al., 2009). SCAP have been observed to have elevated telomerase activity, increased ability to survive infection, a higher rate of population doubling, and superior migratory behavior within the canal space (Sonoyama et al., 2006; Huang et al., 2010). Therefore, SCAP serve as suitable candidates for the regeneration of the pulp-dentin complex.

### Design Criteria for Endodontic Biomaterials

The variable clinical success associated with traditional apexification procedures in teeth with immature roots has recently driven a shift toward the regeneration of the pulp-dentin complex in the field of endodontics research. RET is the process of delivering dental stem cells to the root canal space and aims to reform the pulp-dentin complex to replace compromised dental tissues and allow root development to continue. A clinically-effective regenerative protocol using functional biomaterials would promote further root development and consistently result in the formation of new dentin by the deposition of calcified tissue to increase both root thickness and length, strengthening the tooth against fracture and improving its stability in the dental alveolus.

Multiple clinical, biological and physical factors must be considered when developing biomaterials for endodontic applications. Importantly, clinical compatibility is a fundamental requirement for all materials used in dental procedures. For a biomaterial to be of practical use in endodontic therapies, it should be endogenous or prefabricated and ready-to-use, stored in sterile packaging, adaptable to the eccentric shapes and sizes of root canals, easily manipulated in operatory settings and involve minimal patient discomfort. Short setting times, together with cervical sealability with MTA and antiseptic properties to help ensure and maintain canal sterility are also desirable attributes.

From a biological viewpoint, a suitable scaffold that can support the survival and differentiation of SCAP should mimic the physical and biochemical microenvironment of the root canal. These include growth factors and other bioactive molecules being presented in a spatial-temporally appropriate manner. It should also contain the appropriate extracellular matrix that promotes SCAP adhesion and migration thus serving as a template for tissue regeneration (O'Brien, 2011). Scaffolds that incorporate growth factors, such as TGF- β1, BMP-2, VEGF, and PDGF, further support odontogenic differentiation and drive pulpal revascularization (Zein et al., 2019). Numerous requirements must be considered when selecting an appropriate scaffold to support SCAP survival and proliferation, including: (i) biocompatibility; that is, the material supports SCAP viability and odontogenic differentiation and biodegrades to products that do not cause harm to the host; (ii) architecture with adequate, controllable porosity to permit cell migration, vascularization, as well as the diffusion of nutrients and waste; (iii) mechanical strength suited to the location and anatomy of the afflicted tooth; and (iv) biodegradability such that mature cells may completely replace the scaffold (O'Brien, 2011; Chang et al., 2017). Although hard tissue formation is a goal of RET, the formation of dentin is preferable to cementum due to dentin's higher mineral content and superior physical properties that enhance resistance against fracture. Lastly, to be clinically feasible, an endodontic biomaterials should not be cost prohibitive to patients nor oral health professionals.

Endodontic biomaterials may also be developed in pre-formed or injectable varieties. Pre-formed scaffolds have definite conformations that remain constant when fixed in the target location, while the compliant nature of injectable scaffolds permits their molding to exactly match the unique anatomy of the scaffold's destination. The fluidity of injectable scaffolds offers a number of advantages over pre-formed scaffolds in the context of pulp-dentin regeneration, including their critical ability to occupy and adapt to the irregular topology of the root canal space, their ease of application which reduces patient discomfort, and their capacity to be mixed with SCAP prior to being injected which facilitates exposure to signaling molecules, cell adhesion and the initiation of other downstream processes (Chang et al., 2017). However, cell-free endodontic biomaterials that recruit endogenous cells into the canal space remain more clinically practical, as they avoid clinical hurdles such as regenerative cell isolation, banking and insertion in the root canal that are yet to be routine in dental practice.

### SCAP Scaffolds

The relatively unpredictable clinical outcomes associated with regenerative endodontic procedures have largely been attributed to individual variations in intracanal blood clot formation due to variable sizes of the apical foramen and inconsistencies in the extent of blood influx into the root canal space, which may be compromised by the use of vasoconstrictor-containing local anesthetic (Lenzi and Trope, 2012; Jadhav et al., 2013). Furthermore, varying levels of growth factors and stem cells trapped in the blood clot may influence cell proliferation and odontogenic differentiation, ultimately impacting the degree of root lengthening and thickening in the endodontically treated tooth. Consequently, there has been a recent push to develop strategies to improve the success and predictability of dental tissue regeneration. As with other tissue engineering protocols, the regeneration of the pulp-dentin complex requires a triad of stem cells, growth factors, and a scaffold biomaterial (O'Brien, 2011). Due to the superior tissue-forming properties of SCAP, these cells have recently become a popular focus for regenerative endodontics research endeavors. The interaction of SCAP with highly-porous scaffolds designed to serve as templates for the regeneration of pulpal tissues strongly influences critical stages of reconstruction, including cell adhesion, migration, and proliferation. The recent surge in research investigating intracanal scaffolds and their effects on SCAP specifically has warranted a review of the literature pertaining to this ever-evolving topic. This paper will review the strengths and limitations of various forms of host-derived, naturally-derived, and synthetic scaffolds that have been investigated for pulp-dentin tissue regeneration from SCAP, and will discuss possibilities for future study in scaffold development.

## Previously Investigated Scaffolds for SCAP

Several studies have determined that the introduction of SCAP into the root canal system in the absence of a scaffold inhibits the attachment of viable cells to the canal walls, and thus fails to regenerate the pulp-dentin complex (Trevino et al., 2011; Jadhav et al., 2012). This clearly demonstrates the necessity for a SCAP scaffold to serve as a template for tissue growth in order to have a successful RET. Numerous host-derived, naturally-derived and synthetic scaffolds have been studied for the delivery and growth of SCAP in the root canal space (Table 1). However, a biomaterial that can successfully support guided regeneration of the pulp-dentin complex has yet to be identified.

**Table 1:** Summary of SCAP scaffolds used for regenerative endodontic therapies investigated to date.

**Scaffold**	**Pros**	**Cons**	**References**
Intracanal blood clot	Host compatibility Autologous growth factors Inexpensive Clinical simplicity	Unstable Inconsistent outcomes Bleeding challenges Inadequate mechanical strength	Jadhav et al., 2012 Chrepa et al., 2017 Dianat et al., 2017
Platelet-rich plasma	Host compatibility Autologous growth factor abundance Elevated revascularization rates Inexpensive	Blood collection difficulties Composition variability Rapid growth factor reduction Inadequate mechanical strength Complexity in clinical formation	Trevino et al., 2011 Jadhav et al., 2012 Bezgin et al., 2015 Fernandes and Yang, 2016
Alginate	Biocornpatibility Low immunogenicity Mild gelation requirements Inexpensive Optimal structure for nutrient exchange	Reduced SCAP viability Inadequate mechanical strength Potential pathogen transmission Product variability	Lambricht et al., 2014 Zhang et al., 2013
Hyaluronic acid	Promotes odontogenic differentiation Biocompatibility Biodegradable Bioactive Porous architecture Adapts to canal morphology Pro-angiogenic degradation factors Fast setting	Inadequate mechanical strength Exogenous growth factors Hypersensitivity to bacterial impurities Reduced SCAP viability Formation of reparative dentin only	Ferroni et al., 2015 Pardue et al., 2014 Friedman et al., 2002
Chitosan	Improved SCAP viability Improved odontogenic differentiation Biocompatibility Biodegradable Low cytotoxicity break Low immunogenicity Broad-spectrum antibacterial properties Mechanical strength	Complex gelation scheme and controlled degradation profile	Chang et al., 2017 Shrestha et al., 2016 Souto et al., 2016
PLLA NF-MS with BMP-2	Adapts to canal morphology Biodegradable Exogenous growth factor incorporation Drug incorporation Optimal structure for nutrient exchange	Disorganized tissue formation Lack intrinsic signaling abilities Prohibitive cost	Wang et al., 2016 Horst et al., 2012 Ceccarelli et al., 2017
	Improved odontogenic differentiation Minimally invasive		
PLGA- PEG nanoparticles	Biodegradable Fast setting Low toxicity Biocompatibility Low immunogenicity Anti-fouling Accelerates bone repair	Prohibitive cost Lack intrinsic signaling abilities	Shiehzadeh et al., 2014 Chang et al., 2017
VitroGel 3D with SDF-lα and BMP-2	Biodegradable Adapts to canal morphology Low immunogenicity Low cytotoxicity Improved odontogenic differentiation Mimics pulp ECM Exogenous growth factor incorporation	Prohibitive cost Lack intrinsic signaling abilities	Xiao et al., 2019

Current regenerative endodontic procedures typically utilize intracanal blood clots, as previously described, or platelet-rich plasma (PRP) to form host-derived scaffolds (Chrepa et al., 2017). While these scaffolds supply the necessary signaling molecules and growth factors for tissue regeneration, their use has been complicated by the unpredictable nature of clot formation and challenges in acquiring PRP, as well as limited efficacy (He et al., 2009; Chrepa et al., 2017). To provide a more predictable alternative to host-derived scaffolds, numerous naturally-derived biomaterials have been developed for the delivery of SCAP to the root canal space and tissue regeneration (Chang et al., 2017). These scaffolds include alginate, hyaluronic acid and its derivates, and chitosan. Naturally-derived scaffolds offer several advantages, such as signaling molecules that aid in cell recognition and adhesion (Chang et al., 2017). However, the use of natural-derived products is limited by the possibility of pathogen transmission, foreign body response, poor mechanical properties, and product variability (O'Brien, 2011; Chang et al., 2017).

Several synthetic scaffolds have been studied as potential candidates for the regeneration of the dentin-pulp tissue from SCAP, and are primarily in the form of hydrogels (Chrepa et al., 2017). Synthetic scaffolds that have been investigated for SCAP delivery include nano-fibrous microspheres, hydrogels and PLGA-PEG nanoparticles. These biomaterials avoid the risk of transmitting pathogens, induce a desirable immune response, and can have a consistent production processes that ensure properties such as mechanical strength, porosity, and rate of biodegradation are uniform (Chang et al., 2017; Chrepa et al., 2017). However, synthetic scaffolds lack the intrinsic signaling abilities of naturally-derived scaffolds and have high costs resulting from their complex production (Chang et al., 2017). This section will identify and discuss the advantages and limitations of the host-derived, naturally-derived, and synthetic scaffolds in pre-formed or injectable designs for SCAP delivery and growth that have been investigated to date.

### Host-Derived Scaffolds

#### Intracanal Blood Clot

As previously described, the induction of bleeding and formation of an intracanal blood clot is a current procedure used in regenerative endodontics to provide a scaffold for pulp-dentin regeneration, presumably from SCAP (Chrepa et al., 2017). In immature teeth with open apices, induced bleeding results in the delivery of SCAP from the periradicular tissues of the tooth into the root canal space through the apical foramen, thus eliminating the need to inject foreign stem cells (Trevino et al., 2011). Induced bleeding also allows endogenous hemostatic factors to enter the canal space and form a fibrin clot that supports processes required for SCAP survival and growth. The advantages of an intracanal blood clot are that it provides an autologous scaffold consisting of cross-linked fibrin that contains the growth factors necessary to support SCAP migration, differentiation, vascularization and tissue regeneration, and does not induce a foreign body response (Jadhav et al., 2012; Chrepa et al., 2017; Dianat et al., 2017). These qualities, in addition to the low cost, clinical simplicity, short setting time and cervical sealability with MTA provide an attractive treatment option for both patients and dental practitioners.

Challenges that complicate the use of intracanal blot clots include their instability and unpredictable clinical outcomes as a consequence of unregulated stem cell entry into the canal space, as well as difficulties in invoking bleeding and hemostasis in some patients (Dianat et al., 2017). These obstacles are major limitations of the use of blot clots in regenerative endodontics, and have driven research efforts for more consistent, effective scaffolds. However, given the extremely favorable and clinically-feasible properties of the intracanal blot clot, investigating strategies to improve its reliability may secure this scaffold as the gold standard for RET.

#### Platelet-Rich Plasma-Based Therapeutics

Platelet-rich plasma (PRP) represents an autologous injectable scaffold that has been used in numerous *in vitro* and clinical studies, in both regenerative endodontics and other surgical tissue regeneration procedures (Torabinejad and Turman, 2011; Trevino et al., 2011; Jadhav et al., 2012; Bezgin et al., 2015). A volume of peripheral blood can be obtained from the patient undergoing the endodontic procedure and is mixed with anticoagulants in a test tube. The tube is then spun in a centrifuge to separate the platelets and leukocytes from erythrocytes, which collect at the bottom more rapidly due to their higher density (Saucedo et al., 2012). The PRP is then separated from platelet-poor plasma, and is further processed to increase the platelet concentration up to 1 million/μL, which is approximately 5 times higher than the physiologic platelet concentration (Trevino et al., 2011; Jadhav et al., 2012; Saucedo et al., 2012). The final volume of PRP and platelet concentration varies with the type of preparation system used. Coagulation may be achieved by combining the PRP with saline solution, calcium chloride and bovine thrombin and injecting the mixture into the canal space, waiting 10 min for clot formation. Alternatively, PRP can be carried to the canal space in a collagen sponge, which activates the platelets and enables degranulation (Trevino et al., 2011).

An elevated number of platelets results in a larger overall quantity of growth factors release by degranulation increasing SCAP growth and proliferation rates and expediting the tissue regeneration process (Jadhav et al., 2012; Bezgin et al., 2015). These growth factors include PDGF, TGF-β, insulin-like growth factor (IGF), VEGF, epidermal growth factor (EGF), and epithelial cell growth factor (ECGF), which all aid in the stimulation of revascularization and increase cell proliferation (Trevino et al., 2011). These are critical elements of tissue regeneration and contribute to the appeal of the PRP scaffold. Importantly, it has been hypothesized that the clinical success observed with PRP scaffolds is due to the role these growth factors play in attracting stem cells located in the periapical region, such as SCAP, and facilitating their migration to the root canal space (Torabinejad and Turman, 2011).

Benefits of PRP include elevated rates of angiogenesis and revascularization, which are fundamental for a successful RET. Furthermore, PRP is an attractive scaffold because of its avoidance of a foreign body response and pathogen transmission, and its cost-effective application relative to synthetic scaffolds as well as cervical sealability (Jadhav et al., 2012; Bezgin et al., 2015). A recent clinical study by Ulusoy et al. (2019) evaluated radiographic changes in root dimensions in 88 necrotic human incisors following the treatment using blood clots, PRP, platelet-rich fibrin and a platelet pellet. The study found similar outcomes among all treatment groups, with all teeth scoring high treatment success score in periapical healing, radiographic root development and positive responses to sensitivity tests after an average of 28.25 months. Minor differences in linear measurements of radiographic canal area and radiographic root area were observed when follow-up time was not used as a factor. An important finding in this study is that the injection of a PRP scaffold yields similar clinical outcomes as intracanal blood clots, while presenting less of a risk of root canal obliteration by avoiding the induction of an apical bleed. However, use of the PRP scaffold is limited by the necessity of obtaining blood from pediatric patients who may not comply with the blood collection process, the additional equipment and reagents required to process PRP in-clinic, variability in its composition, and the failure of this scaffold to guide complete, long-term pulp-dentin regeneration, as growth factors are rapidly released upon degranulation and levels significantly decline as the regeneration process proceeds (Trevino et al., 2011; Bezgin et al., 2015; Fernandes and Yang, 2016).

### Naturally-Derived Polymeric Scaffolds

#### Alginate

Alginate is a natural polysaccharide that is purified from the cell walls and intracellular spaces of brown seaweed, and has been extensively used in biomaterial applications (Venkatesan et al., 2014). Alginate hydrogels are formed by crosslinking the polysaccharides with divalent cations to form ionic bridges in a water-insoluble network (Lambricht et al., 2014). Stem cells may be seeded into the gels during this process, which are then injected into the canal space where the gelation process occurs. This rapid gelation feature as well as good mixing properties with other biopolymers have also contributed to the widespread use of alginate as a component and many 3-D printed scaffolds. For example, alginate can be mixed with dentin matrix extracts in equal mass ratio has been fashioned into bioink with high dimensional stability and supports odontoblast-like cells viability (>80%) over a period of 5 days in culture (Athirasala et al., 2018).

The popularity of alginate scaffolds in tissue engineering endeavors can be attributed to its biocompatibility, favorable immunogenicity, low cost, and mild gelation requirements (Zhang et al., 2013). Furthermore, the highly organized, macroporous form of the alginate scaffold permits nutrient/waste exchange and solute diffusion. However, in addition to general complications of natural biomaterials such as potential pathogen transmission, product variability and inadequate mechanical strength, a study conducted by Lambricht et al. (2014) determined that SCAP viability was markedly reduced when exposed to an alginate hydrogel relative to other naturally-derived hydrogels *in vitro*, and the highest levels of apoptosis *in vivo*. Therefore, scaffolds containing only alginate may have limited potential in regenerative endodontic procedures with SCAP. Careful design and blending with other bioactive polymers and growth factors should be considered to extend the usefulness of alginate.

#### Hyaluronic Acid and Derivatives

Several recent studies have investigated the use of hyaluronic acid (HA) as a potential scaffold for SCAP delivery and growth in the root canal space (Lambricht et al., 2014; Chrepa et al., 2017). HA is a glycosaminoglycan consisting of alternating D-glucuronic acid and N-acetyl-D-glucosamine units, which are natural components of the extracellular matrix (ECM) that can interact with SCAP membrane receptors such as CD44, activating signaling pathways that drive cellular migration which could be critical to SCAP recruitment to the root canal space (Lambricht et al., 2014). In the ECM, HA maintains extracellular spacing and, thus, preserves the matrix's morphology (Inuyama et al., 2010). Furthermore, HA has been found in the dental pulp and decreases as teeth develop during odontogenesis, suggesting that HA may have a role in the initial formation of the dentin matrix and pulp (Ferroni et al., 2015). These properties, together with HA's potential to be structurally and chemically modified for a wide range of applications, are of particular interest in the field of dental tissue engineering (Neilson et al., 2015).

HA and its derivatives have numerous advantages, including their biocompatibility, biodegradability, and bioactivity, and their porous architecture that resembles the native pulp-dentin ECM (Chang et al., 2017). HA is often in the form of an injectable fluid that undergoes gelation *in situ*. As such, HA-based scaffolds are able to adapt to the morphology of the root canal and have a relatively fast setting time, which are clinically-attractive features (Ferroni et al., 2015). An analysis completed by Pardue et al. (2014) also suggested that HA degradation products may include pro-angiogenic growth factors, which are instrumental in the revascularization of the regenerated dental tissues (Pardue et al., 2014). Limitations of HA-based scaffolds include their relatively low mechanical strength their requirement to be combined with growth factors such as BMP-2 and TGF-β1 for desirable regeneration of the pulp-dentin complex. Hypersensitivity reactions due to bacterial impurities is another potential complication of HA scaffolds (Friedman et al., 2002).

Several HA derivatives, including HA-based hydrogels like Corgel™ and Restylane, have been investigated as candidates for SCAP delivery and growth (Lambricht et al., 2014; Chang et al., 2017; Chrepa et al., 2017). An *in vitro* study investigating the effects of increasing concentrations of NaOCl on SCAP survival and differentiation in organotype root canal models employed a HA hydrogel as the scaffold, and found that HA alone supported SCAP viability and odontogenic differentiation, both of which were further promoted by the addition of 17% EDTA (Martin et al., 2014). As observed by Chrepa et al. (2017), the HA-based hydrogel Restylane has the ability to increase SCAP mineralization and odontogenic differentiation based on significantly increased alkaline phosphatase activity after seven days in an *in vitro* 3D transwell system compared to scaffold-free controls, and elevated expression of odontoblastic differention markers dentin sialophosphoprotein (DSPP), dentin matrix acidic phosphoprotein 1 (DMP-1) and matrix extracellular phophoglycoprotein (MEPE) after 14 days. However, this scaffold induced the formation of reparative dentin instead of tubule-forming dentin, and significantly compromises a cell viability relative to scaffold-free controls. Lambricht et al. (2014) investigated another HA-based hydrogel, Corgel™, and observed that this scaffold positively impacted SCAP proliferation and metabolism *in vitro*, while increased collagen production and decreased rates of apoptosis were observed *in vivo* when hydrogel and SCAP mixtures were injected into peritoneal pockets in mice. However, similar to Restylane, Corgel™ significantly reduced the degree of SCAP viability after 7 days. As such, HA scaffolds and their derivates serve as possible candidates for regenerative endodontic procedures, however, further investigation is required to improve SCAP viability with this scaffold.

#### Chitosan Derivatives

Chitosan is a linear, cationic aminopolysaccharide biopolymer that is similar to components of the ECM and is produced through *N*-deacetylation of chitin, the main component of the exoskeleton of crustaceans such as crabs and shrimp (Feng et al., 2014; Shrestha et al., 2016). Chitosan also possess reactive amine groups that can aid in the functionalization of bioactive molecules, and can be degraded through enzymatic and hydrolytic reactions to non-cytotoxic metabolites (Jung et al., 2015; Shrestha et al., 2015). Chitosan can be easily molded into a highly-porous structure at a low cost, facilitating processes such as cell migration. Alternatively, chitosan can be prepared in the form of nanoparticles for tissue regeneration through ionotropic gelation (Souto et al., 2016). For the purpose of tissue regeneration, the geometric features of nanoparticles are generally desirable due to the increased surface area for cell adhesion and biological activity compared to other biomaterials formats. Their mass transport properties can be tuned and developed into controlled-release platform of essential growth factors such as TGF-β1, which is critical to support and regulate stem cell differentiation in tissue regeneration procedures (Shrestha et al., 2014). As such, nanoparticles have become an attractive delivery system for bioactive molecules in recent years (Lee et al., 2011).

Advantages of chitosan include its biocompatibility, biodegradability, low cytotoxicity, low immunogenicity, and its broad-spectrum antibacterial properties (Shrestha et al., 2016; Souto et al., 2016; Chang et al., 2017). Furthermore, chitosan nanoparticles are mechanically strong, are resistant to degradation by bacterial enzymes, and have been shown to improve SCAP adhesion, viability, and differentiation, even in environments that have been exposed the powerful root canal antimicrobial agent, NaOCl (Shrestha et al., 2016). However, chitosan use is complicated by its complex gelation and degradation scheme due to its unusual polycationic chain and highly-crystalline structure, thus limiting the range of its potential applications as an injectable scaffold in its naturally-occurring form (Chang et al., 2017).

The usefulness of chitosan nanoparticle as a bioactive, controlled-release scaffold has been demonstrated by Shrestha et al. (2014) where they evaluated SCAP mineralization by measuring alkaline phosphatase (ALP) activity in the presence of chitosan nanoparticles loaded with bovine serum albumin (BSA), a model protein for drug delivery, *in vitro*. Two forms of chitosan nanoparticles were synthesized for use in this study, including BSA-encapsulated (BSA-CSnpI) and BSA-absorbed nanoparticles (BSA-CSnpII). The individual effects of chitosan nanoparticles and BSA on SCAP were simultaneously evaluated. It was observed that BSA-CSnpI showed a 10% release within 10 days, while BSA-CSnpII demonstrated a rapid 40% release within the same time period. At the end of a 3-week trial, BSA-CSnpI demonstrated significantly higher ALP activity than both BSA-CSnpII and CSnp, suggesting that nanoparticles encapsulating BSA best supported SCAP differentiation to hard tissue-forming cells. Researchers speculated that this phenomenon may have been due to the prolonged release of BSA by BSA-CSnpII over the 3-week period, demonstrating the critical importance of the controlled-release of bioactive molecules in SCAP differentiation.

A subsequent *in vitro* study conducted by the same team investigated the same forms of chitosan nanoparticles in dexamethasone-encapsulated (DEX-CSnpI) and dexamethasone-absorbed (DEX-CSnpII) varieties (Shrestha et al., 2015). Unlike the above described BSA-loaded nanoparticle system, rapidly-releasing DEX-CSnpII significantly enhanced odontogenic differentiation compared to slow-releasing DEX-CSnpI and chitosan nanoparticles alone over a 3-week period, based on alizarin red staining for mineralization and real-time reverse-transcription polymerase chain reaction for alkaline phosphatase, DSPP and DMP-1. Shrestha et al. then studied human dentin slabs conditioned with slowly-releasing and rapidly-releasing variants of dexamethasone-releasing chitosan nanoparticles. It was found that rapid-releasing DEX-CSnpII further improved SCAP adhesion and viability relative to unconditioned controls based on calcein-AM staining, and expression of odontogenic differentiation markers DSPP and DMP-1 was markedly increased in DEX-CSnpII conditions compared to DEX-CSnpI as well as unconditioned and CSnp controls after 2 weeks in an immunofluorescent analysis (Shrestha et al., 2016). Importantly, this study determined that CSnp, DEX-CSnpI, and DEX-CSnpII may have the ability to minimize the loss of SCAP viability and adherence in root canal systems that have been disinfected with NaOCl (Trevino et al., 2011; Shrestha et al., 2016).

A carboxymethyl chitosan-based scaffold (CMCS) with TGF-β1-releasing chitosan nanoparticles (TGF-β1-CSnp) was investigated by Bellamy et al. *in vitro*, and significantly enhanced SCAP migration through transwell membranes in 24 h, as well as expression of odontogenic differentiation markers DSPP and DMP-1 compared to scaffolds with TGF-β1 or CSnp alone (Bellamy et al., 2016). Although the definite role of TGF-β1 requires further investigation, several studies have indicated that TGF-β1induces the cytological and functional differentiation of odontoblasts in animal dental papillae cultures, and plays an important role in the secretion of the dentin matrix (Begue-Kirn et al., 1994; Li et al., 2011). The CMCS scaffold is water soluble, compatible with SCAP, and modifies the surface of the dentin matrix such that its antibacterial properties are improved and its ultrastructure is stabilized, hence its use in the Bellamy et al. study (Bellamy et al., 2016). The research team concluded that the CMCS scaffold containing TGF-β1-CSnp enhanced SCAP viability, migration and odontogenic differentiation, suggesting that this system may be yet another promising chitosan-based scaffold for SCAP in RET.

### Synthetic Scaffolds

#### PLLA Nanofibrous Microspheres

The use of poly (L-lactic acid) (PLLA) nanofibrous microspheres (NF-MS) has recently been studied by Wang et al. (2016) as a novel injectable scaffold for SCAP growth. The PLLA NF-MS was evaluated as a SCAP carrier for delivery in combination with poly (lactic-co-glycolic acid, PLGA) microspheres for controlled bone morphogenic protein 2 (BMP-2) release to promote SCAP differentiation into odontoblast-like cells. A chiral isoform of polylactic acid (PLA), PLLA-based scaffolds maintain their integrity for a 42-day period and are, therefore, well-suited for tissue regeneration procedures (Horst et al., 2012). The morphogenic factor BMP-2 had been shown to induce odontogenic differentiation of other dental stem cells *in vitro* and *in vivo* prior to the study by Wang et al., and therefore, was considered a promising candidate for the induction of human SCAP odontogenesis. PGLA is a copolymer formed through the union of polylactic acid (PLA) and polyglycolic acid (PGA) through ester bonds (Ceccarelli et al., 2017). The 12:13 combination of PLA and PGA results in a biomaterial that has an extended half-life relative to both acids individually, increasing the degradation time of the PLGA microspheres and prolonging the exposure of SCAP to BMP-2 (Ceccarelli et al., 2017).

Advantages of PLLA NF-MS with controlled BMP-2 release include their injectability and ability adapt to root canal morphology, biodegradability, and potential for growth factor and drug incorporation. With similar architecture to collagen, high porosity and a large surface area, NF-MS facilitate cell adhesion, growth, as well as nutrient and waste exchange (Wang et al., 2016). The controlled release BMP-2 from PLGA microspheres has been observed to increase SCAP odontogenic differentiation and dentin-like tissue production *in vivo*. Clinically, this would minimize the need to reapply BMP-2 and would thus, reduce the invasiveness of the scaffold's application and minimize the need for complex manipulations (Wang et al., 2016). Like other synthetic scaffolds used in tissue engineering, the PLLA NF-MS system allows for consistency in properties such as the morphology and diameter of the pores and surface features, as well as a low likelihood of inducing a foreign body response (Ceccarelli et al., 2017). However, Wang et al. noted several limitations of the NF-MS scaffold that may comprise its clinical success, such as the disorganized formation of dentin-like tissues because the architecture of the scaffold did not guide the formation of the desired dentinal tubules found in the natural state of the tooth. The cost associated with the complex production of the NF-MS material that incorporates BMP-2 may also be clinically prohibitive, compared to endogenous or naturally-derived scaffolds with innate bioactive capacities. It should also be noted that the degradation of PLLA as well as PLGA release acidic residues into the surrounding microenvironment which may be reduce local cell viability. For this reason, careful control of hydrolytic degradation rate is necessary for i*n vivo* applications.

#### PLGA-PEG Nanoparticles

Recently, Poly (lactide-co gylcolide)-polyethylene glycol (PLGA-PEG) nanoparticles have been clinically investigated as a scaffold for SCAP by Shiehzadeh et al. (2014). PEG is an absorption-resistant polyether with a high molecular weight (Ceccarelli et al., 2017). Together with PLGA, this scaffold has been found to be more conducive to dental pulp fibroblast proliferation and development of dental tissues compared to hydrogel and alginate scaffolds (Shiehzadeh et al., 2014). To determine if PLGA-PEG nanoparticles had similar effects on SCAP, Shiehzadeh et al. seeded this scaffold with autologous SCAP from banked teeth in this study prior to injecting the mixture into patients' root canal system (Shiehzadeh et al., 2014). Periapical healing and was monitored radiographically for 18–24 months postoperatively for each patient.

Compared to many biomaterials, PLGA-PEG nanoparticles have numerous advantages in SCAP scaffold applications. The nanoparticles biodegrade within clinically-feasible time periods (weeks/months) to carbon dioxide and water, and they are in a transparent fluid-form at room temperature that is quickly converted to an opaque gel at 37°C (Shiehzadeh et al., 2014). PLGA-PEG nanoparticles also have a low toxicity, excellent biocompatibility and are minimally immunogenic (Chang et al., 2017). Additionally, the PEG component has an anti-fouling property that inhibits the adherence of residual bacteria to the biomaterial's surface (Chang et al., 2017). In the study conducted by Shiehzadeh et al. (2014), the PLGA-PEG scaffold did not have adverse effects on the tissues surrounding the afflicted tooth, and accelerated periapical bone repair within 6 months while the tooth remained functional. However, like other synthetic scaffolds employed in tissue engineering, the use of the PLGA-PEG nanoparticles scaffold is limited by the clinically-prohibitive costs of production and standardization, as well as the necessity to bank and pre-mix autologous SCAP with scaffolds prior to injection. Furthermore, there was minimal radiographic evidence of continued root formation in length and canal wall thickness in the cases presented by Shiehzadeh et al., suggesting that the injectable PLGA-PEG scaffold may induce apexification while facilitating periapical healing.

#### VitroGel 3D®

The synthetic polysaccharide hydrogel, VitroGel 3D, was recently evaluated as a potential injectable SCAP scaffold by Xiao et al. (2019) *in vitro* and *in vivo*. The synergistic effects of stromal cell-derived factor-1α (SDF-1α) and BMP-2 together with the VitroGel 3D solution on SCAP was also investigated. In this study, VitroGel 3D solution was diluted 1:2 with dionized water before being mixed with SCAP suspension. Hydrogel experimental groups consisted of VitroGel 3D alone and supplemented with SDF-1α and/or BMP-2, each at concentrations of 100 ng/ml. SCAP viability and proliferation were evaluated *in vitro* after 4 days using live/dead staining and cell counting kit (CCK)-8 assays, while odontogenic differentiation was evaluated at three, seven and 14 days using real time RT-PCR, ALP activity and western blot assays. Target genes and proteins included DMP-1 and DSPP, among several other markers of odontogenic/osteogenic differentiation. Finally, the odontogenic differentiation was assessed *in vivo* through ectopic subcutaneous injection in mice after 8 weeks using the same experimental groups.

The results of the study showed that the VitroGel 3D hydrogel did not significantly impact SCAP viability or proliferation compared to 2D controls. Furthermore, SDF-1α and BMP-2 synergistically enhanced the expression of odontogenic genes and proteins *in vitro*, with significantly greater DMP-1 and DSPP expression after 14 days in hydrogels supplemented with both SDF-1α and BMP-2, as well as hydrogels with BMP-2 alone. Histologic and immunohistochemical evaluations of specimens cultured *in vivo* echoed the *in vitro* results, with elevated levels of DSPP expression, osteoid dentin and vascularization in VitroGel 3D with both SDF-1α and BMP-2, as well as with BMP-2 alone, compared to control groups and SDF-1α alone. These results suggest that VitroGel 3D injectable scaffolds may hold promise in supporting intracanal hard tissue deposition and continued root development in RET. Like other injectable scaffolds, however, VitroGel 3D maintains practical issues such as the necessity to bank SCAP and associated expenses currently impede the clinical feasibility of this scaffold.

## Potential Scaffolds for Future Studies

### Scaffold Properties for SCAP Delivery

Existing literature suggests that the type of scaffold selected presents profound influences on critical aspects of the regeneration of the pulp-dentin complex, including SCAP viability, migration, adhesion, differentiation, and mature structural form (Zhang et al., 2013; Wang et al., 2016; Chang et al., 2017). The physical form of the scaffold, as an endogenous substance, pre-formed material, or injectable foam or gel also influences SCAP development pathways and the morphogenesis of the mature dental tissues (Zhang et al., 2013; Wang et al., 2016; Chang et al., 2017). A clinically-relevant scaffold for pulp-dentin tissue regeneration must enable angiogenesis and the vascularization of the regenerated tissue; the organized formation of odontoblast-like cells on existing dentin structures in the root canal; and the coordinated addition of dentin produced by these cells to existing dentin tubules (Huang et al., 2010). Further research is required to develop an effective biomaterial for SCAP delivery and growth in the root canal system. Investigating the application of biomaterials and their derivatives that have been used in other bone regenerative procedures and in protocols that utilize other forms of dental stem cells may prove to be constructive.

### Scaffolds in Other Tissue Regeneration Procedures

As a consequence of the relatively recent identification of SCAP, a more extensive spectrum of scaffolds has been researched for dental pulp stem cells (DPSC), another type of dental stem cell capable of regenerating the pulp-dentin complex *in vivo* (Huang et al., 2009). Although SCAP comprise a post-natal stem cell population that is distinct from DPSC and have a superior dentin regeneration potential, the two stem cell varieties have displayed comparable trends in viability, migration and differentiation potentials when exposed to identical conditions (Sonoyama et al., 2006; Huang et al., 2010; Bakopoulou et al., 2011). Therefore, investigating biomaterials that have successfully guided dentin formation from DPSC progenitors may be worthwhile in future SCAP scaffold research.

Scaffolds that have been studied for DPSC delivery and growth but have yet to be investigated for SCAP applications include collagen type-I, collagen type-III, gelatin, silk protein, and peptide scaffolds including self-assembling peptides and peptide amphiphiles (Zhang et al., 2013; Gong et al., 2016). Of these, collagen, self-assembling peptide scaffolds such as Puramatrix™, and calcium polyphosphate have experienced significant success and will be discussed (Cavalcanti et al., 2013; Gong et al., 2016).

#### Collagen

Collagen is a natural biomaterial that is widely-used in tissue regeneration applications as a result of its architectural resemblance of many tissues' extracellular matrix and its ability to adapt to the morphology of the target (Zhang et al., 2013). Type I collagen is the most abundantly used, and best promotes DPSC proliferation and mineralization capacity compared to other collagen types (Gong et al., 2016; Chang et al., 2017). A pre-formed collagen sponge scaffold has been studied with success for the delivery of DPSC to the root canal system *in vivo* (Sumita et al., 2006). Additionally, a recent clinical study completed by Nosrat et al. (2019) evaluated the use of SynOss™ putty, a bovine type I collagen and synthetic carbonate apatite material, as an intracanal scaffold in three human patients with non-infected immature first premolars scheduled for extraction. After 2.5–7 months, teeth treated with SynOss™ putty together with an intracanal blood clot displayed histologic evidence of mineralized, cementum-like tissues on dentinal walls, while teeth treated with SynOss™ putty alone displayed asymptomatic periapical lesions radiographically with no new intracanal tissue, and intracanal blood clots alone resulted in the formation of fibrotic connective tissue with malformed cementum in the canal space, along with reparative cementum on dentinal walls. These findings suggest that type I collagen-based scaffolds together with dental stem cell-containing blood clots may promote intracanal hard tissue formation compared to blood clots alone.

Advantages of collagen include its biocompatibility and bioactivity, owing to its motifs that can be recognized by DPSC which facilitate adhesion and downstream signaling pathways for proliferation and differentiation, as well as its extracellular matrix that is structurally comparable to that of the pulp-dentin complex (Kim et al., 2009; Zhang et al., 2013; Chang et al., 2017). The porous structure of collagen also facilitates its colonization by seeded stem cells (Sumita et al., 2006). However, the difficulties encountered in collagen scaffold studies pertaining to regenerative endodontics are its low mechanical strength, irregular biodegradation and the generation of tissues that resemble connective tissue instead of dentin *in vivo* (Kim et al., 2009). Furthermore, like other naturally-derived scaffolds, product variability and risk of immunogenicity and pathogen transmission complicate the clinical applicability of collagen scaffolds.

#### Self-Assembling Peptide Hydrogel: Puramatrix™

Puramatrix™ is a synthetic, self-assembling peptide hydrogel that creates a 3D environment that is biocompatible, biodegradable, and non-toxic to cells (Aligholi et al., 2016). The hydrogel exists as an aqueous solution, however, polymerizes to form a solid gel instantly when exposed to physiologic salt conditions and is, thus, a clinically-practical scaffold (Nune et al., 2013).

In a study of DPSC seeded in a Puramatrix™ scaffold conducted by Cavalcanti et al. (2013), it was observed that Puramatrix™ enabled DPSC viability and proliferation *in vitro*, as well as odontoblastic differentiation over a 3-week period when DPSC and Puramatrix™ were seeded on tooth slices. Contrary to other primary cell types, varying concentrations of Puramatrix™ did not significantly affect DPSC proliferation; a 0.2% gel was, therefore, employed because of its increased rigidity and stability. It was speculated in this study that the soluble factors responsible for odontogenic differentiation diffused from existing dentin in the tooth slice models that were employed in the study, since odontogenic differentiation was not observed in DPSC cultured with Puramatrix™ in the absence of tooth slices. An additional *in vitro* and *in vivo* study by Dissanayaka et al. (2015) investigated Puramatrix™ together with DPSC alone and co-cultured with human umbilical vein endothelial cells (HUVEC). Similar to Cavalcanti et al. (2013) and Dissanayaka et al. (2015) found that the Puramatrix™ scaffold supported DPSC survival, while DPSC/HUVEC co-cultures demonstrated significantly higher viability than either monoculture. Furthermore, significantly elevated ALP expression and greater mineralization was observed in DSPC/HUVEC co-cultures compared to DSPC monocultures after seven days. Histological evaluation of human tooth root segments loaded with each cell group and Puramatrix™ after 4 weeks *in vivo* in mice revealed similar patterns, with osteodentin formation adjacent to dentin in DPSC/HUVEC co-cultures together with dentin sialoprotein (DSP) expression, as well as significantly greater vascularization an extracellular matrix deposition in the co-culture compared to other groups. Based on these promising findings, it can be speculated that Puramatrix™ may also prove to be a suitable scaffold for SCAP in regenerative endodontic applications.

#### Calcium Polyphosphate/Calcium Phosphate Cement

Calcium polyphosphate (CPP) scaffolds have been extensively studied in bone repair and regenerative applications due to their biocompatibility, controllable degradability, mechanical strength and similarity to naturally-occurring bone (Xie et al., 2016). As with other inorganic polyphosphates, CPP serves as a source of phosphate that induces bone differentiation in osteoblasts (Ozeki et al., 2015). The scaffold also has a chain-like structure with oxygen atoms connecting monomeric subunits that provide easily-accessible sites for hydrolysis to naturally-occurring, readily-metabolized calcium orthophosphate products (Comeau et al., 2012). As the CPP scaffold degrades, released calcium, and phosphorous components contribute to the formation of calcified tissues, such as dentin (Maruyama et al., 2016).

A study completed by Wang et al. (2006) investigated the viability of human DPSC when exposed to a porous CPP scaffold *ex vivo*. The scaffold was fabricated from a mixture of porous agent and amorphous powder, which was seeded with DPSC. The porous CPP scaffold allowed for effective nutrient/waste exchange, had no cytotoxic effect on the DPSC, improved cell adhesion and migration, and had no adverse effects on proliferation. A subsequent study conducted by Ozeki et al. (2015) observed that polyphosphate induced matrix metalloproteinase (MMP)-3 expression in purified odontoblast-like cells derived from pluripotent stem cells from mice. The MMP-3 expression increased cell proliferation and resulted in increased expression mature odontoblastic phenotype markings, including DMP-1 and DSPP. It can be speculated that CPP may exhibit similar effects on SCAP differentiation to odontoblasts-like cells. Because of the comparable behaviors of DPSC and SCAP, a CPP scaffold may exert similar influences on SCAP and could be an effective biomaterial in RET.

Calcium phosphate cement (CPC) has also been investigated as a scaffold for human DPSC (Qin et al., 2018). Although CPC alone has relatively weak strength properties, the incorporation of chitosan has been shown to increase flexural strength of CPC-based scaffolds (Weir and Xu, 2010). Qin et al. (2018) loaded CPC with 15% liquid chitosan and 50 μg of metformin, and evaluated DPSC viability and proliferation after 7 days, as well as odontogenic differentiation, ALP activity and mineralization after 14–21 days when combined with the scaffolds *in vitro*. Live/Dead and CCK-8 assays demonstrated that the CPC scaffold supports DPSC viability and proliferation, while ALP activity and mineralization were significantly increased in CPC with both chitosan and metformin, as was expression of odontoblastic gene markers, including DSPP, DMP-1, Runt-related transcription factor 2 (RUNX2), and OCN (osteocalcin) mRNA determined by quantitative RT-PCR. These findings suggest that, like CPP, CPC may also support SCAP growth and differentiation based on this material's effects on DPSC.

## Conclusions

Current research relating to SCAP delivery, growth, and differentiation shows a great deal of promise for future clinical applications of these cells in regenerative endodontic procedures. Although a variety of scaffolds have been developed for SCAP delivery and growth in the root canal space, a candidate that meets all the requirements for an functional scaffold in dental tissue regeneration remains to be identified. Scaffolds derived from host, natural and synthetic source each possess desirable features as well as disadvantages that limit their clinical feasibility. This paper has outlined the beneficial properties and limitations of biomaterials that have been previously developed as SCAP scaffolds, and has described potential scaffolds for future investigation based on their performance with DPSC in aspiration of drawing nearer to a consistent, clinically-successful RET.

## Author Contributions

GR, IM, and BL contributed to the writing and editing of this manuscript.

### Conflict of Interest

The authors declare that the research was conducted in the absence of any commercial or financial relationships that could be construed as a potential conflict of interest.
